# Early detection of mental health on social media using a hybrid Bi-LSTM–XGBoost model: a comparative study

**DOI:** 10.1038/s41598-026-47015-6

**Published:** 2026-05-04

**Authors:** Heru Syah Putra, Muhammad Ary Murti, Astri Novianty, Nur Hafieza Ismail

**Affiliations:** 1 Electrical Engineering Study Program, School of Electrical Engineering, Telkom University, 40257 Bandung, Indonesia; 2 Computer Engineering Study Program, School of Electrical Engineering, Telkom University, 40257 Bandung, Indonesia; 3 Faculty of Computing, University Malaysia Pahang Al-Sultan Abdullah, Pekan Pahang, 26600 Malaysia

**Keywords:** Mental health detection, NLP, Twitter, Social media, Boosting, Diseases, Health care, Mathematics and computing, Psychology, Psychology

## Abstract

The case of mental health disorders has been a main topic in the clinical and psychological field. The advancement of computing studies, especially in Natural Language Processing (NLP)—a subset of Machine Learning, created a system of detection that can detect the mental health state of a person in early stage to prevent the eventuality of the worst case. This is crucial since there has been a lot of case of mental health disorder—such as depression and suicide, remains undetected and untreated–especially when the internet usage is more prevalent than ever even among the most vulnerable users, which are the preadolescent users. This study explores the models that can accurately predict mental health disorder with the provided six labels the model can predict. The labels are anxiety, depression, personality disorder, stress, bipolar, and normal. The dataset is gathered from a Kaggle repository which is then processed and refined further for the training process. From multiple evaluations across diverse amount of texts from different users, our Bi-LSTM-XGBoost model outperforms the other models with an accuracy of 0.9035 and 0.4320 loss, while other models fall short within 50–84% accuracy. Further improvement can be made with our model, whether from improving the model’s parameters further or by improving the quantity and quality of the dataset gathered.

## Introduction

Mental health disorders such as depression, anxiety, bipolar, and addictive behavior continue to constitute a public health crisis with social^1^ and economic consequences^2^. Early detection is crucial, as delay is associated with worsening of the condition and an increased risk of suicide. The creation of a real-time monitoring strategy is therefore important to speed up psychological intervention. Recent research shows that language footprints on social networks reflect current mood, stress, and symptomatic indicators in real life^3,4^. These indicators, which may include an increase in the use of negative lexicons, a decrease in positive affection, and other behavioral posting patterns, can be mapped for early detection of a low-cost and non-intrusive physiological status^5^.

From a sociological or clinical psychology perspective, the problematic use of social networks acts as a coping mechanism and is correlated with several internal disorders, such as sleep-related disorders, low self-esteem, and others. Research also links the intensity of social media use with the elevated risk of substance abuse, body image over-concern, and eating disorders^6^. These findings reinforce the urgency of developing models that can predict the risk of addiction from online behavioral data^7^.

Within the domain of Natural Language Processing (NLP) in model computation, deep learning approaches have surpassed the classical methods to classify psychological symptoms from social media texts, especially when given an unbalanced data set^8^. Transformer-based and LSTM-based models consistently performed greatly in detecting mental disorders such as depression and suicide, especially when tuned with linguistic features and attention mechanisms. Currently, there is a growing emphasis on the need for explainability in the model to ensure that the output can be used and interpreted by professional clinicians easily.^9^.

Current literature shows common limitations, especially when many currently studied models only trained on binary or four diagnostic classification labels, such as depressed with normal^10,11^; and rely solely on a singular algorithm. This is in contrast with the broader spectrum of mental health disorders that necessitate a broader and more representative multi-label classification. Furthermore, the linguistic complexity of social media language that can be characterized by the length of text, slang, and sarcasm–demands models that are robust to contextual nuances and ambiguities^12^.

Building on this foundation, this current research focuses on the early detection of mental health. A Bi-LSTM-XGBoost model is used and compared with other four different algorithms across five different distinct labels simultaneously. This multi-label approach is designed to push greater generalization and enhancement of clinical relevance compared to a regular binary classification scenario. This design is expected to produce a model characterized by higher sensitivity and specificity, improve explainability, and be easily translatable for integration into community or clinical services^13^.

## Theory

Interaction of Person-Affect-Cognition-Execution (I-PACE) model conceptualizes addiction, including problematic social media use, as the outcome of interactions between predisposing factors such as traits, affective-cognitive processes (e.g. desire thinking, cue reactivity, and craving), and executive control dysfunctions^14^. These interactions develop progressively through the initiation of the behavior to its potential relapse. This model has been updated and generalized across various non-substantial addictive behaviors, thus its relevancy is shown to map the addiction risk of online digital platforms or social media such as X (Twitter)^15^. In its most recent elaboration, desire thinking is positioned as the cognitive mechanism that mediates the engagement of compulsive behavior, related to inhibitory control modulation and craving within the digital addiction landscape.

From the perspective of Uses & Gratifications (U&G), motives related to information exploration, entertainment, and escapism intensify the usage of social media across all ages. This is particularly a problem for the individual with poor self-control, leading to psychological distress that cycles through a consistent pattern of social media use^16^. Social psychology added that social comparison on social media can lead to upward comparison, decrease in body satisfaction, increase in stress, and consumption of impulsive contents, shown by daily bi-directional mapping of risk dynamics in near real-time, which is relevant in preemptive detection in this case^6,11^.

**Table 1:** Previous research in the last 5 years.

No	Title	Author	Publisher	Model	Dataset	Results
1	An attention-based CNN-BiLSTM model for depression detection on social media text	Thekkekara et. al	Elsevier, 2024	CNN + Bi-LSTM	CLEF2017	96.71%
2	Diagnosis of Depression Based on Four-Stream Model of Bi-LSTM and CNN From Audio and Text Information	Jo et. al	IEEE, 2022	CNN + Bi-LSTM	EATD-Corpus	97%
3	Mental Health: Detection & Diagnosis	Parimala et. al	IEEE, 2022	Logistic Regression	Self-created questionnaires	92%
4	Detection of major depressive disorder, bipolar disorder, schizophrenia, and generalized anxiety disorder using vocal acoustic analysis and machine learning: an exploratory study	Espinola et. al	Springer, 2022	Random Forest	SRQ-20, HAM-D 17, BPRS, YMRS and GAD-7	90.01%
5	Mental illness detection using sentiment analysis in social media	Odja et. al	Elsevier, 2024	Random Forest	Zenodo	80.65%

Previous studies, summarized in Table 1, show that our proposed architecture attempts to address a number of remaining theoretical gaps. Most previous studies relied solely on binary classification models or a limited number of labels (fewer than five), so that complex addiction and distress profiles were often simplified and did not reflect the diversity of real-world contexts^17,18^. Multi-modal research focusing on audio-text combinations or single algorithms reports high accuracy, but is generally constrained by limited labeling schemes, thereby reducing the generalization of results^19^. On the other hand, approaches based solely on sentiment analysis tend to lose important information related to the specificity of mental disorders that are more subtle and profound^20^. In addition, a number of studies that produce minority classes often face problems of model instability and limitations in terms of interpretability^18,21^. Thus, our contribution is aimed at overcoming these limitations through a more comprehensive and reliable architectural design. From studying and analyzing the previous research studies, an advancement can be made–which, in our research, the Bi-LSTM-XGBoost model is used as the chosen model for the detection task.

Within the psycholinguistic field, language-based signal such as negative emotions, self-references, sarcasm, or slang syntax pattern, is strongly linked with depression, anxiety, suicide ideation, and addictive burdens. Lexicon approaches such as Linguistic Inquiry and Word Count (LIWC) or modern neural networks prove to be quite effective on parsing social media data^8^.

Recent studies are now pushing more with a multi-labeled model to represent the wide spectrum of mental health conditions (e.g., depression, anxiety, stress, suicide ideation, etc.), in contrast to the binary labeled model. The corpus based on Twitter/X or Reddit enforces a richer labeling scheme and a more general evaluation^22^. In addition to that, explainability is also equally important in health topics that require a reliable explanation, which both the professional and patient can use reliably^23^.

## Methods

This section describes the methodological framework employed in our study, consisting of three main stages: (i) data collection, annotation, and preprocessing, (ii) model representation using a hybrid architecture, and (iii) evaluation of the proposed approach. The methodological pipeline begins with the acquisition of social media data and their preparation through a series of preprocessing steps, followed by structured labeling into multiple psychological risk categories. Subsequently, we design a hybrid model that stacked the sequential learning capabilities of Bi-LSTM with the predictive strength of XGBoost.

**Figure 1:**
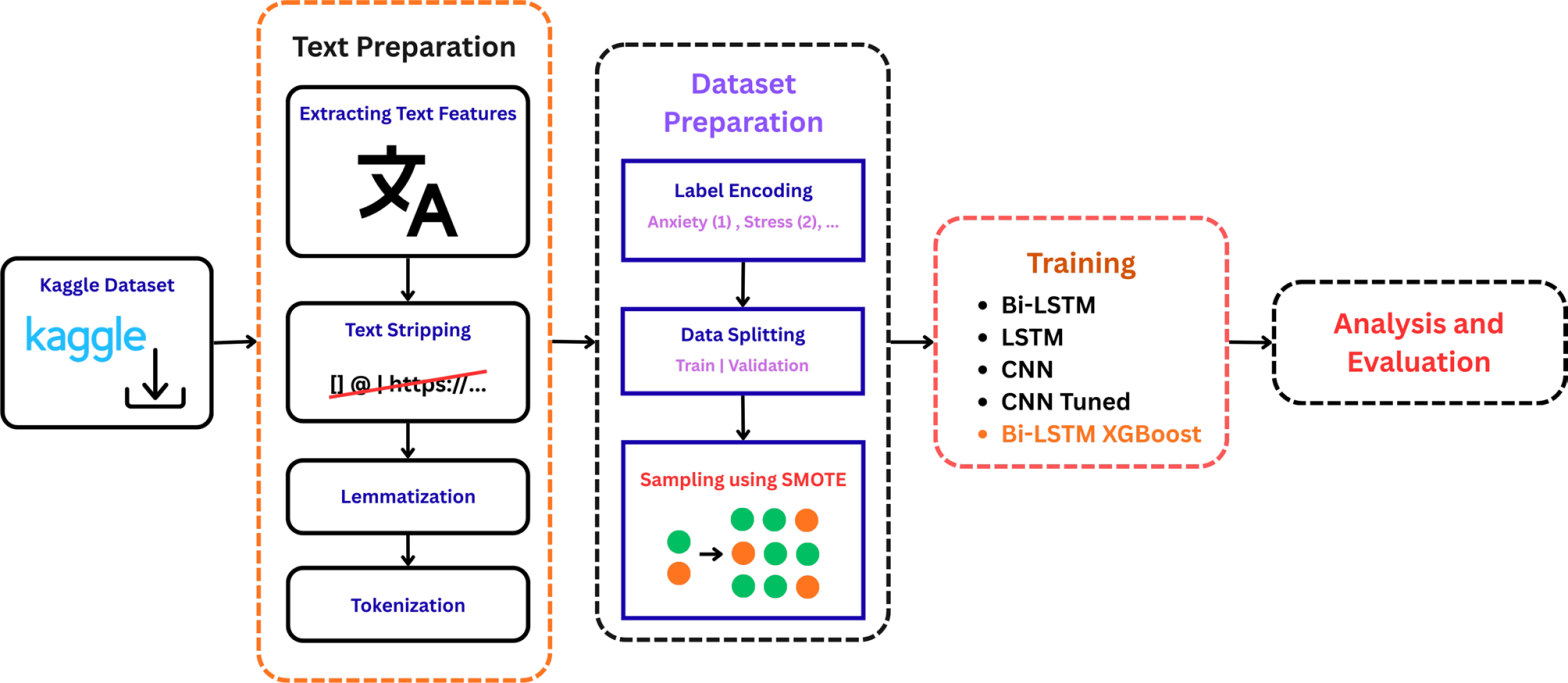
Step of processing in diagram block.

The overview of our training process workflow can be seen in Fig. 1. Following dataset acquisition, text and dataset preparation are done before the training process starts. The detailed description of each component is presented in the following subsections.

### Data collection, annotation, and preprocessing

The research data are obtained through a kaggle repository, specifically from Suchintika Sarkar’s “Sentiment Analysis for Mental Health’, which is relevant for our research purposes of early detection of psychological risk. The retrieved data were subsequently organized into a six-label schema (anxiety, depression, personality disorder, bipolar, stress, and normal), which provides a richer representation of mental states compared to the common practice of using only two to four labels in previous studies. In this experiment, a label from the original dataset is dropped as we discover the majority of the sentences and paragraphs on “Suicidal” label heavily resemble to that of “Depression” label.

The preprocessing step includes URL cleaning, hashtags segmentation, lowercasing, slang/elongation normalization, and lemmatization. Texts are tokenized and represented through word embeddings or contextual embeddings. If *V* is the vocabulary and $f:V \rightarrow Rd$ is the embedding function, then for all token $w_i$ yield a vector equation $x_i = f(w_i), x_i \in \mathbb {R}$. For class imbalance, we incorporate Synthetic Minority Over-sampling Technique (SMOTE) by generating synthetic sample for the minority class instead of deduplicating for existing ones.

### Hybrid model representation: BiLSTM-XGBoost

**Figure 2:**
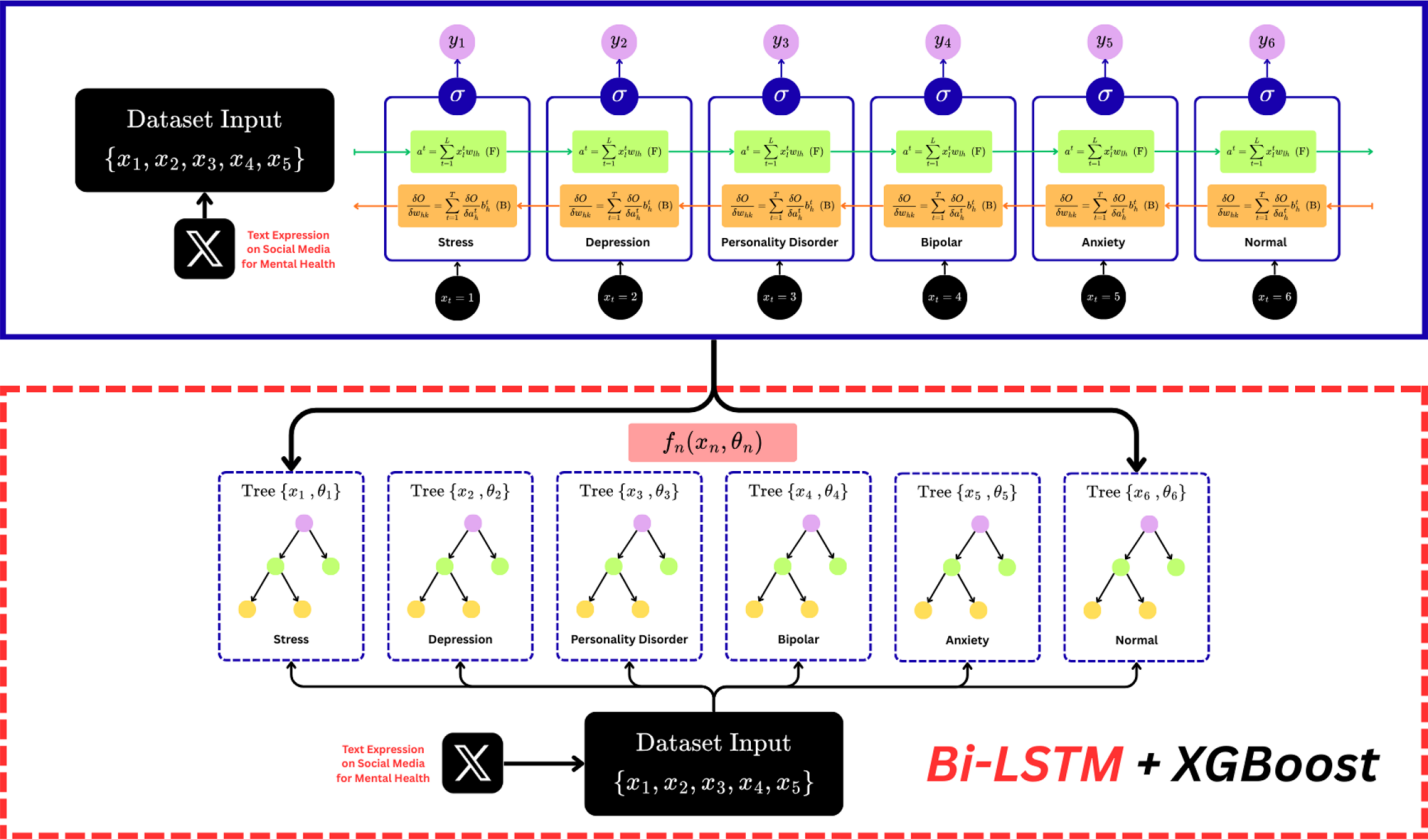
Hybrid model.

In this hybrid architecture, a stacking architecture is utilized to combine the strengths of XGBoost as a discriminative learner with the sequential representation power of Bi-LSTM. The process flow is as follows: First, XGBoost is trained on TF-IDF vectors and tabular numerical features (specifically, the number of characters and the number of sentences) to generate preliminary multi-class prediction probabilities. Concurrently, textual sequences pass through an Embedding layer and a Bi-LSTM network to extract deep contextual features. Finally, the sequential Bi-LSTM features, the tabular numerical features, and the XGBoost prediction probabilities are concatenated and fed into fully connected dense layers to produce the final classification. The block diagram of our design can be seen in Fig. 2.

#### XGBoost: gradient boosting algorithm

XGBoost is a gradient boosting decision tree (GBDT)-based algorithm that works by iteratively optimizing the objective function through the addition of new decision trees that minimize the residual error from the previous iteration. The objective function of XGBoost is formulated as:

1. Initialize model with a constant value $\hat{f} _{(0)} (x) = \arg _{0} \min \sum \limits _{i=1}^{N} L (y_{i}, \theta )$
2. For m = 1 to M:
  - Compute the ’gradients’ and ’Hessians’:
    1 $$\begin{aligned} & \hat{g}_{m}(x_{i}) = \left[ \frac{\partial L (y_i. f(x_i))}{\partial f(x_i)}\right] _{f(x) = \hat{f}_{(m-1)}(x)} \end{aligned}$$
    2 $$\begin{aligned} & \hat{h}_{m}(x_{i}) = \left[ \frac{\partial ^2 L (y_i. f(x_i))}{\partial f(x_i)^2}\right] _{f(x) = \hat{f}_{(m-1)}(x)} \end{aligned}$$
  - Fit a base learner (or weak learner, e.g., tree) using the training set $\lbrace x_i, \frac{\hat{g}_m (x_i)}{\hat{h}_m (x_i)}_{i=1}^N \rbrace$ by solving the optimization problem below:
    3 $$\begin{aligned} & \hat{\phi }_m = \arg _{\phi \in \Phi }\sum \limits _{i=1}^N \frac{1}{2}\hat{h}_m (x_i) \left[ \phi (x_i) - \frac{\hat{g}_m (x_i)}{\hat{h}_m (x_i)}\right] ^2 \end{aligned}$$
    4 $$\begin{aligned} & \hat{f}_m (x) = \alpha \hat{\phi }_m (x) \end{aligned}$$
    Where,
    $$\begin{aligned} & \alpha \text {: Learning Rate} \\ & L(y, F(x))\text {: Differentiable Loss Function} \\ & \left\{ \left( x_i,y_i\right) \right\} _{i=1}^N\text {: Training Set} \\ & M \text {: A number of weak learners} \\ \end{aligned}$$
3. Update the calculated model:
  5 $$\begin{aligned} \hat{f}_m (x) = \hat{f}_{m-1}(x)-\hat{f}_m(x) \end{aligned}$$

In our hybrid framework, XGBoost acts as a foundational predictor whose output probabilities are fed into the input alongside the Bi-LSTM features and tabular data. In our experiment, the following configuration is used: learning rate of 0.1, maximum depth of 7, *n* estimators of 500, random state of 101, and the objective is multi-soft probability.

#### Bi-LSTM: forward–backward mechanism and unit design

Bidirectional Long Short-Term Memory (Bi-LSTM) is an extension of the standard LSTM architecture that processes sequential data in both forward and backward temporal directions, enabling the model to capture long-range dependencies from past and future contexts simultaneously. The fundamental LSTM unit was originally introduced to address the vanishing gradient problem in recurrent neural networks^24^, while the bidirectional formulation further enhances sequence modeling by incorporating contextual information from both directions^25^.

Given an input sequence $\{x_t\}_{t=1}^T$, a Bi-LSTM consists of two independent LSTM layers: a forward LSTM that computes the hidden state $\overrightarrow{h}_t$ by processing the sequence from $t=1$ to *T*, and a backward LSTM that computes $\overleftarrow{h}_t$ by processing the sequence from $t=T$ to 1. The final Bi-LSTM representation at time step *t* is obtained by concatenating the forward and backward hidden states:

6 $$\begin{aligned} h_t = \left[ \overrightarrow{h}_t \, ; \, \overleftarrow{h}_t \right] \end{aligned}$$

The combined hidden state $h_t$ is then passed to a softmax layer to produce the multi-class probability distribution:

7 $$\begin{aligned} p_t = \text {softmax}(W_o h_t + b_o) \end{aligned}$$

Both forward and backward LSTM units follow the standard gating mechanism. At each time step *t*, the LSTM computes the input gate, forget gate, output gate, and candidate cell state as follows:

8 $$\begin{aligned} i_t&= \sigma (W_i x_t + U_i h_{t-1} + b_i) \end{aligned}$$

9 $$\begin{aligned} f_t&= \sigma (W_f x_t + U_f h_{t-1} + b_f) \end{aligned}$$

10 $$\begin{aligned} o_t&= \sigma (W_o x_t + U_o h_{t-1} + b_o) \end{aligned}$$

11 $$\begin{aligned} \tilde{c}_t&= \tanh (W_c x_t + U_c h_{t-1} + b_c) \end{aligned}$$

The cell state and hidden state are updated according to:

12 $$\begin{aligned} c_t&= f_t \odot c_{t-1} + i_t \odot \tilde{c}_t \end{aligned}$$

13 $$\begin{aligned} h_t&= o_t \odot \tanh (c_t) \end{aligned}$$

where $\sigma (\cdot )$ denotes the sigmoid activation function, $\tanh (\cdot )$ is the hyperbolic tangent function, and $\odot$ represents element-wise multiplication.

#### Hybrid integration and training procedure

To enhance clarity and reproducibility, the workflow of the proposed hybrid framework is summarized in Algorithm 1. The pseudocode outlines the key steps of model construction, beginning with the initialization of sequential and tabular inputs, followed by the Bi-LSTM network and the XGBoost classifier. These components are then concatenated and refined through dense layers with regularization, leading to the final softmax output for multi-class psychological risk detection.

**Algorithm 1:**
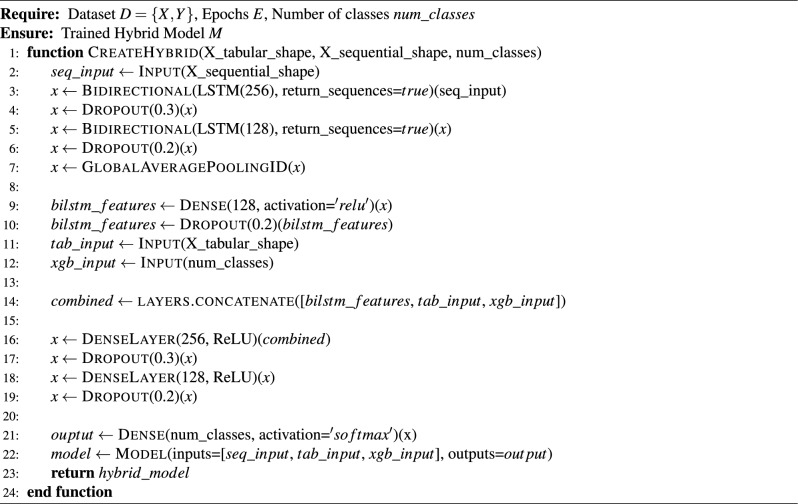
Hybrid Model

The key implementations for the model are as follows: Lemmatization is used for tokenization, after which the dataset is split into train and validation sets. Next, numerical tabular features are extracted from each text, specifically the number of characters and the number of sentences. The text data is then vectorized through a TF-IDF Vectorizer and combined with the numerical features using a horizontal stack. SMOTE is applied to the training set to tackle class imbalance.

The embedding size of our model is 512, with max sequence length of 100 and learning rate of 0.00001 within 30 epochs across all model. Additionally, the batch size used for training is 64. The tabular data is the train data that has already processed in SMOTE used for extra information for Bi-LSTM and XGBoost model. Early stopping is not used in this experiment.

To train the model, we first run the XGBoost model on the resampled training dataset to extract prediction probabilities. Then, we create the hybrid stacking architecture with sparse categorical cross-entropy as the loss function and Adam as the optimizer. Finally, we train the hybrid model by passing the reshaped sequential data, the SMOTE-resampled tabular data, and the XGBoost prediction probabilities as simultaneous inputs to predict against the validation labels.

For the other model, we used this following configuration for the comparison: With Bi-LSTM, we use 2 layers of Bi-LSTM with 256 and 128 each which then pass through GlobalAveragePoolingID, and then finally goes through 3 dense layer of 256, 128, and the final of 7 layers. The same approach are used for LSTM, but without the bidirectional part. For CNN, we first reshape the input of train originally for LSTM to be compatible with the CNN model, then using Conv1D with value of 16 up to 256 for the tuning process. Dropout is then used with the value of 0.3 which then pass through GlobalMaxPoolingID. The final layer is the dense layer, with the softmax value of 7.

## Experimental results

### Experimental dataset

The experimental dataset was obtained through Suchintika Sarkar’s “Sentiment Analysis for Mental Health” repository, yielding 42,029 text samples. The distribution includes 16,343 labeled as normal, 15,404 as depression, 3,841 as anxiety, 2,777 as bipolar, 2,587 as stress, and 1,077 as personality disorder. To ensure robust evaluation of the proposed hybrid Bi-LSTM–XGBoost framework, we utilized an 80/20 train-validation split ratio. Specifically, 33,624 text samples were used for the training set, and the remaining 8,405 samples were maintained strictly as a validation set. No separate held-out test split was used; all performance metrics, tables, and figures reported in this study reflect the model’s performance on this 20% validation set. Prior to training, preprocessing such as text cleaning, tokenization, and normalization was performed to ensure data consistency.

The repositories contains contents from people all over the world, not limited to just one specific country, with most of the text comes from United States and other countries that have English as the main language. This ensures that during the training process, the model is not skewed into some specific bias that some countries have because of the inherent contextual language difference influenced by the culture of those countries.

### Result and analysis

**Figure 3:**
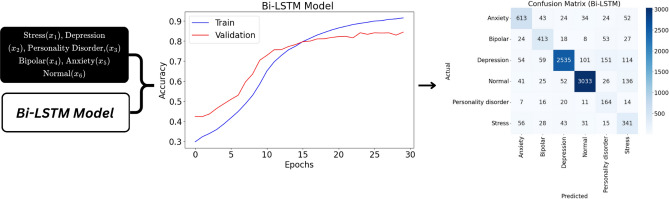
Result Bi-LSTM model accuracy.

**Figure 4:**
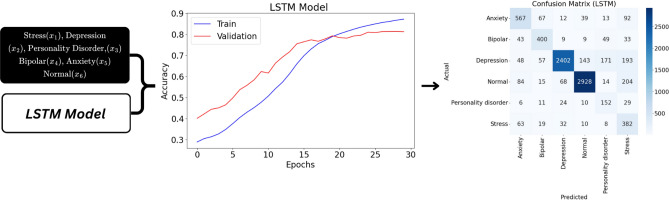
Result LSTM model accuracy.

**Figure 5:**
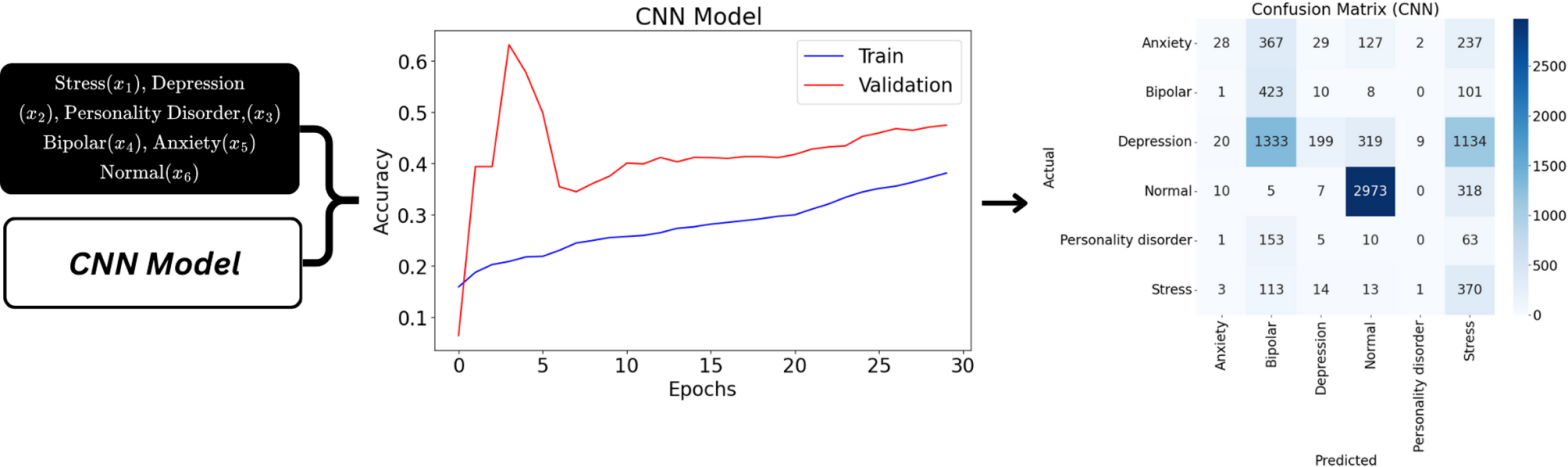
Result CNN model accuracy.

**Figure 6:**
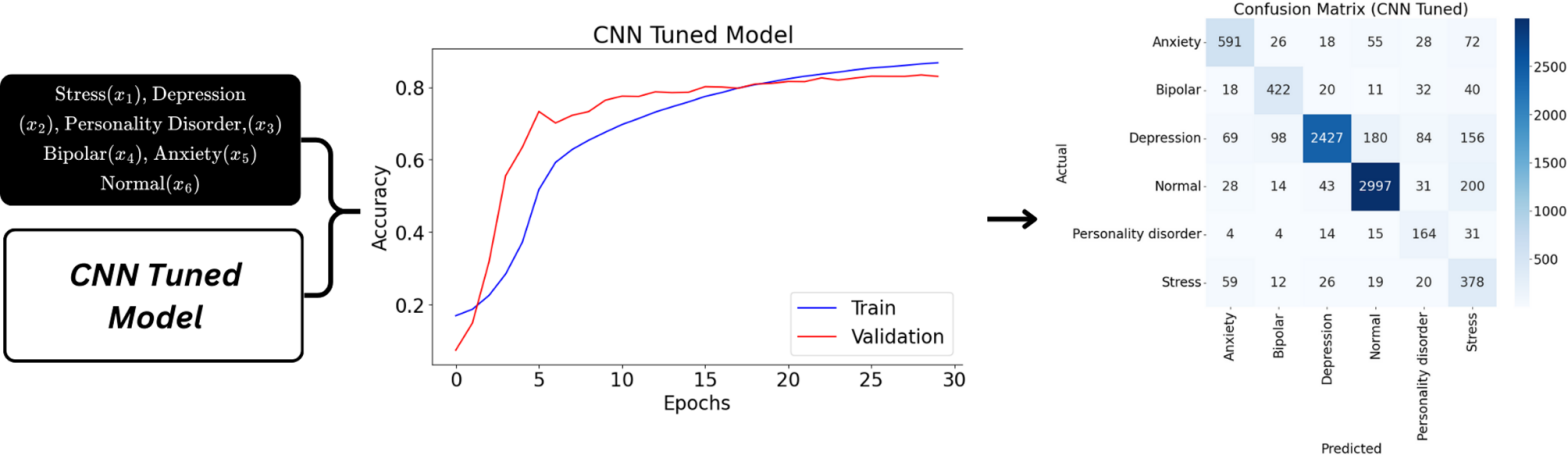
Result CNN (tuned) model accuracy.

**Figure 7:**
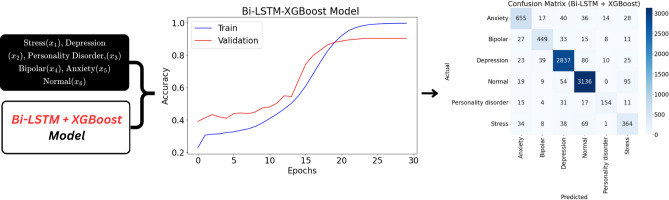
Result Bi-LSTM + XGBoost model accuracy.

In this experiment, we compared our model with 4 different sets of algorithms: Bi-LSTM, LSTM, CNN, and CNN with additional tuning. After the training step, prediction results were gathered and plotted into an epoch graph of train and validation accuracy, where we set the value to 30 for the epochs across all models. From it, we analyze and evaluate which model was able to perform the best in terms of accuracy on the validation set given the restrictions of an imbalanced dataset. The results can be seen in the figures below (Figs. 3, 4, 5, 6, 7).

The comparative analysis across five models–Bi-LSTM, LSTM, CNN, CNN (Tuned), and the proposed Bi-LSTM–XGBoost – reveals clear performance distinctions in detecting mental health risks from social media data across six labels (anxiety, bipolar, depression, normal, personality disorder, and stress). The Bi-LSTM outperformed the standard LSTM by a small margin by capturing richer contextual dependencies from both past and future words in a sentence, which proved particularly beneficial for the classification of nuanced conditions like depression and anxiety.

However, both recurrent models faced significant challenges with minority classes compared to our model, such as stress and personality disorder, a limitation directly attributable to the class imbalance in the dataset which led to a bias towards the more populous “depression” and “normal” categories. The un-tuned CNN model showed limited ability in handling the long-range sequential nuances of language, treating text more locally like a bag-of-features. While its tuned variant achieved quite a gains through hyperparameter optimization (e.g., adjusting filter sizes and dropout rates), it remained less effective.

In contrast, the hybrid Bi-LSTM–XGBoost consistently delivered the best results. This performance boost stems from its stacking architecture: the XGBoost model effectively captures lexical and tabular patterns using its powerful gradient-boosting mechanism, outputting highly discriminative class probabilities. These probabilities are then concatenated with the deep, contextual sequential features extracted by the Bi-LSTM layer. By combining these diverse feature representations in a final set of dense layers, the hybrid model provides a more reliable and balanced framework for the early detection of psychological risks, underscoring its potential applicability in real-world, imbalanced social media datasets.

The use of SMOTE as the balancer also boost the performance of the model. Without SMOTE, the hybrid model only yields 0.6905 accuracy with 0.8735 loss compared to 0.9035 and 0.4320 accuracy and loss respectively with SMOTE.

**Table 2:** Model comparison.

No	Model	Macro avg	Weighted avg	Accuracy	Loss	Epochs
1	Bi-LSTM	0.73	0.85	0.8418	0.5319	30
2	LSTM	0.70	0.83	0.8148	0.5875	30
3	CNN	0.27	0.43	0.5082	1.0663	30
4	CNN (Tuned)	0.83	0.84	0.8323	0.5709	30
$\boxed {5}$	$\boxed {\mathrm{Bi-LSTM + XGBoost}}$	$\boxed {0.83}$	$\boxed {0.90}$	$\boxed {0.9035}$	$\boxed {0.4320}$	$\boxed {30}$

Table 2 provide the final quantitative comparison of the model performances, clearly demonstrating the advantages of our Bi-LSTM–XGBoost hybrid. To confirm even further, we run the test for the hybrid model for 10 times and we found the average of the accuracy to be 0.9023 with the standard deviation of 0.0014. This significant margin in performance, coupled with the lowest loss, underscores the synergy of the hybrid approach.

**Table 3:** F1-score label comparison.

No	Model	Anxiety	Bipolar	Depression	Normal	PD	Stress
1	Bi-LSTM	0.77	0.73	0.89	0.93	0.49	0.57
2	LSTM	0.71	0.72	0.86	0.91	0.48	0.53
3	CNN	0.07	0.29	0.12	0.88	0.00	0.27
4	CNN (Tuned)	0.76	0.75	0.87	0.91	0.55	0.54
$\boxed {5}$	$\boxed {\mathrm{Bi-LSTM + XGBoost}}$	$\boxed {0.84}$	$\boxed {0.84}$	$\boxed {0.94}$	$\boxed {0.94}$	$\boxed {0.74}$	$\boxed {0.69}$

**Table 4:** Recall label comparison.

No	Model	Anxiety	Bipolar	Depression	Normal	PD	Stress
1	Bi-LSTM	0.78	0.76	0.84	0.92	0.71	0.66
2	LSTM	0.72	0.74	0.80	0.88	0.66	0.74
3	CNN	0.04	0.78	0.07	0.90	0.00	0.72
4	CNN (tuned)	0.75	0.78	0.81	0.90	0.71	0.74
$\boxed {5}$	$\boxed {\mathrm{Bi-LSTM + XGBoost}}$	$\boxed {0.83}$	$\boxed {0.83}$	$\boxed {0.94}$	$\boxed {0.95}$	$\boxed {0.66}$	$\boxed {0.71}$

**Table 5:** Precision label comparison.

No	Model	Anxiety	Bipolar	Depression	Normal	PD	Stress
1	Bi-LSTM	0.77	0.71	0.94	0.94	0.38	0.50
2	LSTM	0.70	0.70	0.94	0.93	0.37	0.41
3	CNN	0.44	0.18	0.75	0.86	0.00	0.17
4	CNN (Tuned)	0.77	0.73	0.95	0.91	0.46	0.43
$\boxed {5}$	$\boxed {\mathrm{Bi-LSTM + XGBoost}}$	$\boxed {0.85}$	$\boxed {0.85}$	$\boxed {0.94}$	$\boxed {0.94}$	$\boxed {0.82}$	$\boxed {0.68}$

For the specific performance of each label, it is shown on Tables 3, 4 and 5. Again, Bi-LSTM-XGBoost hybrid outperforms all of the models in each labels. The largest improvements are in Personality Disorder (denoted as PD) and Stress, with almost 20% jump in accuracy. Anxiety and Bipolar labels score a sizable improvement of 10 %, while Anxiety and Normal labels improve by just a small margin. These results solidly validate the hybrid model as the most effective model for this application.

**Table 6:** Hybrid model components comparison.

No	Model	Macro Avg	Weighted Avg	Accuracy	Loss	Epochs
1	Bi-LSTM	0.73	0.85	0.8418	0.5319	30
2	XGBoost on Bi-LSTM features	0.79	0.88	0.8741	0.6227	30
3	Stacking Bi-LSTM + XGBoost	0.83	0.90	0.9035	0.4320	30

Table 6 shows the results of the ablation study conducted to analyze the contribution of each component in the proposed stacking architecture. The Bi-LSTM model serves as the baseline, capturing bidirectional contextual dependencies in the textual data and achieving reasonable performance. Incorporating tabular features (character and sentence counts) into the Bi-LSTM provides an intermediate boost. Finally, when XGBoost predictions are integrated with both the Bi-LSTM–extracted features and the tabular features via the stacking architecture, a noticeable improvement is observed across all evaluation metrics, particularly in macro-average and weighted-average scores. This indicates that the architecture is able to exploit both the structural lexical data from XGBoost and the high-level semantic representations generated by the Bi-LSTM more effectively than a neural classifier alone, especially under class-imbalanced conditions.

### Evaluation

**Figure 8:**
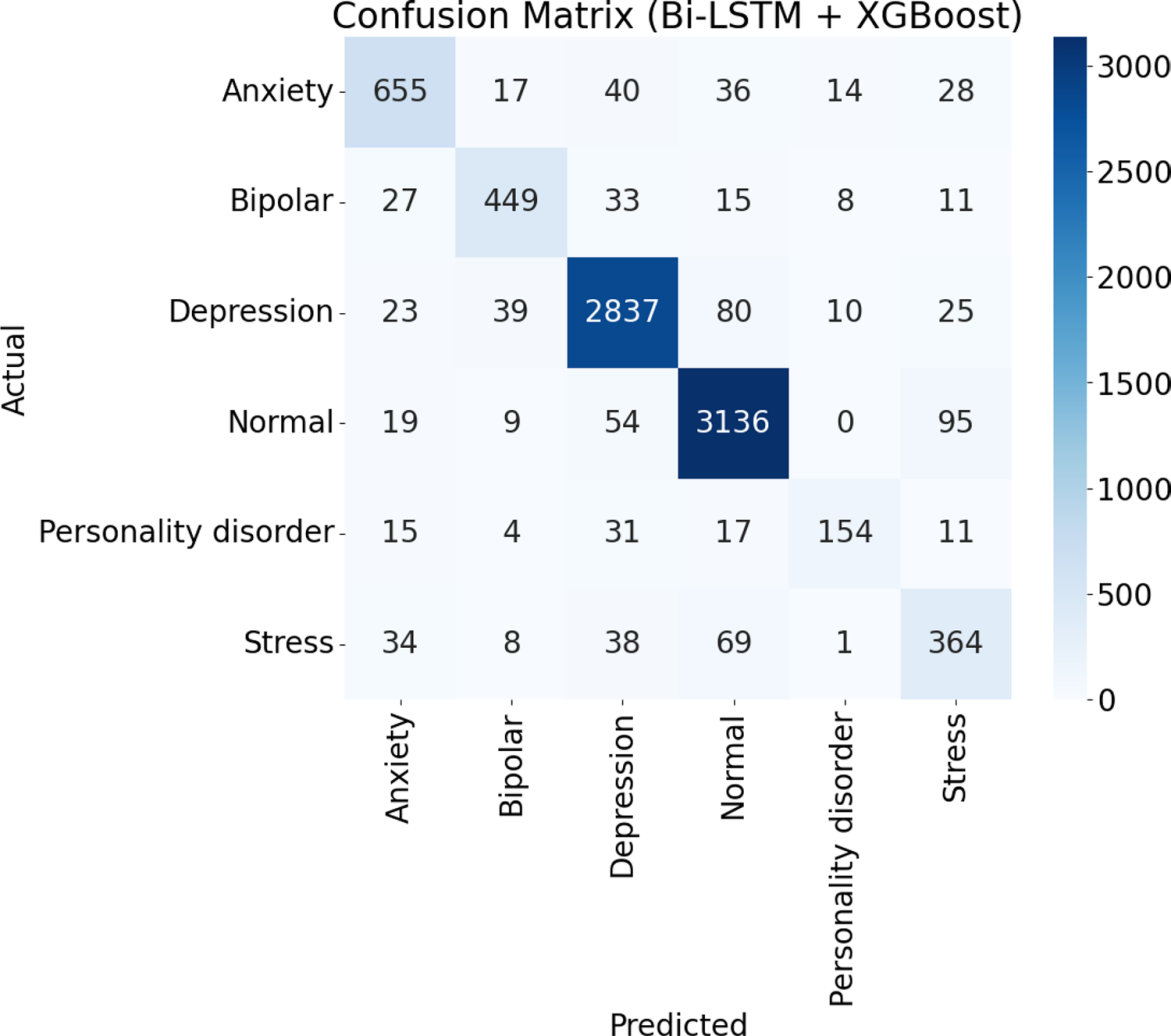
Bi-LSTM + XGBoost confusion matrix.

The evaluation results can be found in Fig. 8. From five randomly picked samples from the validation dataset shown in Table 7, we can see that the model performs exceptionally well even with longer text. It only fails on the second text, where it mistook bipolar as a personality disorder. On the second text, words like “reactive person” and “self-destructive” potentially misled the model to accurately detect Bipolar disorder, since some of the words in some of the mental health disorder’s dictionaries share the same words as in other mental health disorders.

**Table 7:** Example texts for evaluation.

No	Text	Predicted label	True label
1	It will be my first time trying wellbutrin. Are there any side effects? How does it affect sex drive? I am a female. Has anyone tried wellbutrin	Depression	Depression
2	How do you deal with and sit through intense emotion im a reactive person and i usually end up doing something selfdestructive when im feeling something intense it usually not possible for me to talk to somebody or run a bubble bath a selfhelp people would have you do what are some thing you all do to prevent yourself from coping in negative way	Personality disorder	Bipolar
3	Is the place to print like that, still open today, urgent for thesis, hmm Jogja only	Normal	Normal
4	I am here all alone, and nobody seems to care that I am sad every day. I just want someone to talk to	Depression	Depression
5	Hopefully something changes for the better, I have given myself 246 days to get my life in order, or I am going to kill myself. Wish me luck	Depression	Depression

In summary, with an accuracy of 90.35% as established in the previous section, the model proves to be highly accurate and effective for the task of mental health detection from text. It excels at capturing the complex relationship between linguistic patterns and psychological states. While the misclassifications in Sample 2 indicates a limitation in distinguishing between conditions with semantically similar expressions of emotional volatility, the overall performance confirms the model’s practical utility for initial screening and prioritization, where its high accuracy can reliably flag at-risk individuals for further review.

## Discussion

The experimental results demonstrate that the proposed Bi-LSTM–XGBoost hybrid model provides a clear performance advantage over all other architectures. By combining Bi-LSTM’s contextual sequence modeling with XGBoost’s gradient-boosted decision-making, the hybrid framework effectively addresses weaknesses observed in individual models–particularly in handling class imbalance and capturing nuanced linguistic cues present in mental-health–related social media text.

Compared to LSTM and CNN variants, the Bi-LSTM alone performed well but still struggled with minority classes such as personality disorder and stress. XGBoost’s integration substantially improved these categories, yielding up to 20% higher F1-scores, indicating stronger discrimination for less frequent disorders. CNN-based models, even when tuned, remained less effective due to their limited ability to model long-range dependencies, reaffirming the importance of contextual sequence learning in psychological risk detection. Misclassifications, such as confusion between bipolar and personality disorder, highlight the challenge posed by overlapping linguistic features across psychological conditions. This underscores the need for enriched domain-specific text representations or more granular labeling in future work.

## Conclusion

The early detection of mental health disorders cannot be understated to be very crucial as it can be a matter of life of a person. NLP has proven to be the key in accurately detecting mental health disorders. There are still challenges in processing human language data, such as gathering balanced amount of quality dataset with the added problem of handling the inner complexity of modern language. Our Bi-LSTM-XGBoost model serves as a way to ameliorate the imbalance problem with the addition of XGBoost model. Potential improvement can be made with the improvement of more diverse dataset gathering and additional fine-tuning of the model.

## Data Availability

The dataset used for training on this research are publicly available in the Suchintika Sarkar repository on Kaggle, https://www.kaggle.com/datasets/suchintikasarkar/sentiment-analysis-for-mental-health/data.

## References

[1] Kawachi, I. & Berkman, L. F. Social ties and mental health. *J. Urban Health* **78**, 458–467. 10.1093/jurban/78.3.458 (2001). 11564849 10.1093/jurban/78.3.458 PMC3455910

[2] Frank, R. G. & McGuire, T. G. Chapter 16 economics and mental health. vol. 1 of *Handbook of Health Economics*, 893–954. 10.1016/S1574-0064(00)80029-3 (Elsevier, 2000).

[3] Cataldo, I., Billieux, J., Esposito, G. & Corazza, O. Assessing problematic use of social media: where do we stand and what can be improved?. *Curr. Opin. Behav. Sci.* **45**, 101145. 10.1016/j.cobeha.2022.101145 (2022).

[4] Casale, S. Problematic social media use: Conceptualization, assessment and trends in scientific literature. *Addict. Behav. Rep.* **12**, 100281. 10.1016/j.abrep.2020.100281 (2020). 32426449 10.1016/j.abrep.2020.100281 PMC7225612

[5] Khan, A., & Ali, R. Unraveling minds in the digital era: a review on mapping mental health disorders through machine learning techniques using online social media. *Soc. Netw. Anal. Min.* **14**, 10.1007/s13278-024-01205-0 (2024).

[6] Portingale, J., Girardin, S., Liu, S., Fuller-Tyszkiewicz, M. & Krug, I. Daily bi-directional effects of women’s social media-based appearance comparisons, body satisfaction, and disordered eating urges. *J. Eat. Disord.* **12**, 129. 10.1186/s40337-024-01096-8 (2024). 39227958 10.1186/s40337-024-01096-8 PMC11369994

[7] Oprea, S.-V. & Bâra, A. Assessing the dual impact of the social media platforms on psychological well-being: A multiple-option descriptive-predictive framework. *Comput. Econ.* **66**, 10.1007/s10614-024-10717-y (2025).

[8] Chen, H., McKeever, S. & Delany, S. J. A comparison of classical versus deep learning techniques for abusive content detection on social media sites. In Staab, S., Koltsova, O. & Ignatov, D. I. (eds.) *Social Informatics*, 117–133 (Springer International Publishing, Cham, 2018).

[9] Kerasiotis, M., Ilias, L. & Askounis, D. Depression detection in social media posts using transformer-based models and auxiliary features. *Soc. Netw. Anal. Min.* **14**, 196. 10.1007/s13278-024-01360-4 (2024).

[10] Chiong, R., Budhi, G. S., Dhakal, S. & Chiong, F. A textual-based featuring approach for depression detection using machine learning classifiers and social media texts. *Comput. Biol. Med.* **135**, 104499. 10.1016/j.compbiomed.2021.104499 (2021). 34174760 10.1016/j.compbiomed.2021.104499

[11] Kim, J. et al. A systematic review of the validity of screening depression through facebook, twitter, instagram, and snapchat. *J. Affect. Disord.* **286**, 360–369. 10.1016/j.jad.2020.08.091 (2021). 33691948 10.1016/j.jad.2020.08.091

[12] Li, Z. et al. Mha: a multimodal hierarchical attention model for depression detection in social media. *Health Inf. Sci. Syst.* **11**, 6. 10.1007/s13755-022-00197-5 (2023). 36660408 10.1007/s13755-022-00197-5 PMC9846704

[13] Bao, E., Pérez, A. & Parapar, J. Explainable depression symptom detection in social media. *Health Inf. Sci. Syst.* **12**, 47. 10.1007/s13755-024-00303-9 (2024). 39247905 10.1007/s13755-024-00303-9 PMC11379836

[14] Brand, M. et al. The interaction of person-affect-cognition-execution (i-pace) model for addictive behaviors: Update, generalization to addictive behaviors beyond internet-use disorders, and specification of the process character of addictive behaviors. *Neurosci. Biobehav. Rev.* **104**, 1–10. 10.1016/j.neubiorev.2019.06.032 (2019). 31247240 10.1016/j.neubiorev.2019.06.032

[15] Brandtner, A., Antons, S., Cornil, A. & Brand, M. Integrating desire thinking into the i-pace model: a special focus on internet-use disorders. *Curr. Addict. Rep.* **8**, 459–468. 10.1007/s40429-021-00400-9 (2021).

[16] Wei, D., Chan, L.-S., Du, N., Hu, X. & Huang, Y.-T. Gratification and its associations with problematic internet use: A systematic review and meta-analysis using use and gratification theory. *Addict. Behav.* **155**, 108044. 10.1016/j.addbeh.2024.108044 (2024). 38663155 10.1016/j.addbeh.2024.108044

[17] Philip Thekkekara, J., Yongchareon, S. & Liesaputra, V. An attention-based cnn-bilstm model for depression detection on social media text. *Expert Syst. Appl.* **249**, 123834. 10.1016/j.eswa.2024.123834 (2024).

[18] Aggarwal, R. & Goyal, A. Anxiety and depression detection using machine learning. In *2022 International Conference on Machine Learning, Big Data, Cloud and Parallel Computing (COM-IT-CON)*, vol. 1, 141–149, 10.1109/COM-IT-CON54601.2022.9850532 (2022).

[19] Jo, A.-H. & Kwak, K.-C. Diagnosis of depression based on four-stream model of bi-lstm and cnn from audio and text information. *IEEE Access* **10**, 134113–134135. 10.1109/ACCESS.2022.3231884 (2022).

[20] Hossain, M. M. et al. Revolutionizing mental health sentiment analysis with bert-fuse: A hybrid deep learning model. *IEEE Access* **13**, 85428–85446. 10.1109/ACCESS.2025.3568340 (2025).

[21] Odja, K. D., Widiarta, J., Purwanto, E. S. & Ario, M. K. Mental illness detection using sentiment analysis in social media. *Proc. Comput. Sci.* **245**, 971–978, 10.1016/j.procs.2024.10.325 (2024). 9th International Conference on Computer Science and Computational Intelligence 2024 (ICCSCI 2024).

[22] Garg, M., Liu, X., Sathvik, M., Raza, S. & Sohn, S. Multiwd: Multi-label wellness dimensions in social media posts. *J. Biomed. Inform.* **150**, 104586. 10.1016/j.jbi.2024.104586 (2024). 38191011 10.1016/j.jbi.2024.104586 PMC10923126

[23] Rai, S. et al. Key language markers of depression on social media depend on race. *Proc. Natl. Acad. Sci.* **121**, e2319837121. 10.1073/pnas.2319837121 (2024). 10.1073/pnas.2319837121 PMC10998627 38530887

[24] Hochreiter, S. & Schmidhuber, J. Long short-term memory. *Neural Comput.* **9**, 1735–1780. 10.1162/neco.1997.9.8.1735 (1997). 9377276 10.1162/neco.1997.9.8.1735

[25] Graves, A. & Schmidhuber, J. Framewise phoneme classification with bidirectional lstm networks. In *Proceedings. 2005 IEEE International Joint Conference on Neural Networks, 2005.*, vol. 4, 2047–2052 vol. 4, 10.1109/IJCNN.2005.1556215 (2005). 10.1016/j.neunet.2005.06.042 16112549

